# Supplementary material for: Global Distribution of Founder Variants Associated with Non-Syndromic Hearing Impairment
Source: Genes (Basel). 2023 Feb 3;14(2):399. doi: 10.3390/genes14020399 (PMC9957346; doi:10.3390/genes14020399)
Supplement: Supplementary file 1 [file genes-14-00399-s001.zip › Table S1.pdf]

Table S1. Founder variants retrieved characteristics and population distributions

rsID	N	DFN	Founder Variant	Freq. (%)	Age yrs	Origin Population	Europe Ref/Alt	Africa Ref/Alt	Asia Ref/Alt	Ref
rs80338948	29	DFNB1	*GJB2*: c. 427C>T-p.Arg143Trp	NA	1,500	Ghana	G=0.999976 / A=0.000024	G=0.9998 / A=0.0002	G=0.9998 / A=0.0002	[11]
rs80338942	546	DFNB1	*GJB2*: c.167delT-p.Leu56ArgTer26	4.03	NA	Ashkenazi Jews	A=0.99817 / =0.00183	A=0.9994 / =0.0006	A=1.000 / =0.000	[24]
rs80338939	124	DFNB1	*GJB2*: c.35delG-p.Gy12Ter2	NA	10,000	Europe	CCCCCC=0.99298/ CCCCC=0.00702, CCCCCCC=0.00	CCCCCC=0.9991 / CCCCC=0.0009, CCCCCCC=0.00	CCCCCC=1.000 / CCCCC=0.000, CCCCCCC=0.00	[25]
rs80338943	105	DFNB1	*GJB2*: c.235delC (p.Leu79CysTer3)	NA	10,000	Middle East	GGG=1.00/GG=0.00	GGG=1.0000 / GG=0.0000	GGG=0.982 / GG=0.018	[25]
NA	33, [illegible]	DFNB1	*GJB6*: Δ(GJB6-D13S1830)	69.6, 98.1	NA	Jews	None	None	None	[40,41]
rs80338939	273	DFNB1	*GJB2:* c.35delG-p.Gly12Ter2	72.1	NA	West-Austria	CCCCCC=0.99298/ CCCCC=0.00702, CCCCCCC=0.00	CCCCCC=0.9991/ CCCCC=0.0009, CCCCCCC=0.0000	CCCCCC=1.000/ CCCCC=0.000, CCCCCCC=0.000	[26]
rs80338943	87	DFNB1	*GJB2*: c.235delC-p.Leu79CysTer3	NA	11500	Japan	GGG=1.00 / GG=0.00	GGG=1.0000/ GG=0.00	GGG=0.982 / GG=0.018	[36]
rs80338943	122 [illegible]	DFNB1	*GJB2*: c.235delC -p.Leu79CysTer3	13.4	NA	Japan	GGG=1.00/ GG=0.00	GGG=1.00 / GG=0.00	GGG=0.982 / GG=0.018	[37]
NA	371	DFNB1	*GJB2*: delE120	NA	NA	Turkey	N/A	N/A	N/A	[42]
rs80338939	371	DFNB1	*GJB2*: c.35delG-p.Gy12Ter2	NA	NA	Turkey	CCCCCC=0.99298/ CCCCC=0.00702, CCCCCCC=0.00	CCCCCC=0.9991/ CCCCC=0.0009, CCCCCCC=0.000	CCCCCC=1.000 / CCCCC=0.000, CCCCCCC=0.000	[42]
rs80338943	76	DFNB1	*GJB2*: c.235delC-p.Leu79CysTer3	6.67	NA	Altai Republic	GGG=1.00 / GG=0.00	GGG=1.00 / GG=0.00	GGG=0.982 / GG=0.018	[38]
rs80338939	127	DFNB1	*GJB2*: c.35delG-p.Gly12Ter2	7.4	NA	China	CCCCCC=0.99298 / CCCCC=0.00702, CCCCCCC=0.00	CCCCCC=0.9991 / CCCCC=0.0009, CCCCCCC=0.00	CCCCCC=1.000 / CCCCC=0.000, CCCCCCC=0.00	[28]

rs80338943	45	DFNB1	*GJB2*: c.235delC - p.Leu79CysTer3	NA	11500	Mongolia & Caucasians	GGG=1.00 / GG=0.00	GGG=1.00 / GG=0.00	GGG=0.982 / GG=0.018	[36]
rs80338939	30	DFNB1	*GJB2*: c.35delG-p.Gy12Ter2	NA	2 700	Morocco	CCCCCC=0.99298/ CCCCC=0.00702, CCCCCCC=0.00	CCCCCC=0.9991/ CCCCC=0.0009, CCCCCCC=0.0000	CCCCCC=1.000 / CCCCC=0.000, CCCCCCC=0.000	[29]
rs80338939	60	DFNB1	*GJB2*: c.35delG-p.Gy12Ter2	NA	14000	Greece	CCCCCC=0.99298 / CCCCC=0.00702, CCCCCCC=0.00	CCCCCC=0.9991/ CCCCC=0.0009, CCCCCCC=0.0000	CCCCCC=1.000 / CCCCC=0.000, CCCCCCC=0.000	[30]
rs104894396	86	DFNB1	*GJB2*: c .71G>A-p.Trp24Ter	32.5	3480	India	C=0.99999 / T=0.00001	C=1.00/ T=0.00	C=1.00/ T=0.00	[43]
rs80338939	100	DFNB1	*GJB2*: c.35delG-p.Gly12Ter2	21.0	NA	India	CCCCCC=0.99298 / CCCCC=0.00702, CCCCCCC=0.00	CCCCCC=0.9991/ CCCCC=0.0009, CCCCCCC=0.00	CCCCCC=1.000 / CCCCC=0.000, CCCCCCC=0.00	[31]
rs80338939	56	DFNB1	*GJB2*: c.35delG-p.Gy12Ter2	NA	3,300-11,800	Russia	CCCCCC=0.99298 / CCCCC=0.00702, CCCCCCC=0.00	CCCCCC=0.9991/ CCCCC=0.0009, CCCCCCC=0.00	CCCCCC=1.00 / CCCCC=0.00, CCCCCCC=0.00	[32]
rs80338939	50	DFNB1	*GJB2*: c.35delG-p.Gly12Ter2	NA	NA	Iran	CCCCCC=0.99298 / CCCCC=0.00702, CCCCCCC=0.00	CCCCCC=0.9991/ CCCCC=0.0009, CCCCCCC=0.00	CCCCCC=1.00 / CCCCC=0.00, CCCCCCC=0.00	[33]
rs2274084	125	DFNB1	*GJB2*: c.79G>A-p.Val27Ile	15	~22,000	Altai Republic & Mexico	C=0.998225 / T=0.001775	C=0.9964 / T=0.0036	C=0.6808 / T=0.3192	[44]
rs104894413	133	DFNB1	*GJB2*: c.131G>A-p.Trp44Ter	7.89	NA	Guatemala	C=1.0000 / T=0.00	C=1.00 / T=0.00	C=1.00 / T=0.00	[45]
NA	2569	DFNB1	*GJB2*: del(GJB2-D13S175)	NA	3000	Russia	NF	NF	NF	[1]
rs80338939	24	DFNB1	*GJB2*: c.35delG-p.Gy12Ter2	4.1	4800 - 8100	Russia	CCCCCC=0.99298 / CCCCC=0.00702, CCCCCCC=0.00	CCCCCC=0.9991/ CCCCC=0.0009, CCCCCCC=0.00	CCCCCC=1.00/ CCCCC=0.00, CCCCCCC=0.00	[34]
rs80338939	131	DFNB1	*GJB2*: c.35delG-p.Gly12Ter2	59.52	NA	Iran	CCCCCC=0.99298 / CCCCC=0.00702, CCCCCCC=0.00	CCCCCC=0.9991/ CCCCC=0.0009, CCCCCCC=0.00	CCCCCC=1.000 / CCCCC=0.000, CCCCCCC=0.000	[35]
rs80338948	7408	DFNB1	*GJB2:* c.427C>T-p.Arg143Trp	2.28	6500	Japan	G=0.999976 / A=0.000024	G=0.9998 / A=0.0002	G=0.9998 / A=0.0002	[23]
rs1302739538	18	DFNB1	*GJB2:* c.516G>C-p.Trp172Cys	64.3	2275 - 4500	Russia	C=1.00 / T=0.00	C=1.00000 / T=0.00	C=1.00000 / T=0.00	[39]

rs80338943	4	DFNB1	*GJB2*: c.235delC-p.Leu79CysTer3	14.3	1125-3150	Russia	GGG=1.00 / GG=0.000	GGG=1.00/ GG=0.00	GGG=0.982 / GG=0.018	[39]
rs80338943	127	DFNB2	*GJB2*: c.235delC-p.Leu79CysTer3	15.5	NA	China	GGG=1.00 / GG=0.00001	GGG=1.00 / GG=0.00	GGG=0.982 / GG=0.018	[28]
rs1567620939	97	DFNB3	*MYO15A*: c.1171_1177dupGCCATCT-p.Tyr393Cysfs*41	28.0	NA	Oman	None	None	None	[46]
rs749136456	19	DFNB3	*MYO15A*: c.4198G>A-p.Val1400Met	42.1	NA	Brazil	G=1.00/A=0.00, C=0.00	G=1.00/A=0.00, C=0.00	G=1.00 A=0.00, / C=0.00	[47]
rs28942097	258	DFNB6	*TMIE*: c.250C>T-p.Arg84Trp	10.3	625–2500	Southeastern Anatolia	C=0.99996 / T=0.00004	C=1.00 / T=0.00	C=1.000 / T=0.0000	[48]
rs121908073	156	DFNB7/11	*TMC1*: c.100C>T-p.Arg34Ter	7.05	1076 - 1900	Tunisia	C=0.9999 / T=0.0001	C=1.0000 / T=0.0000	C=1.000 / T=0.000	[108]
rs937270834	159	DFNB7/11	*TMC1*: c.-258A>C	2.2	NA	Iran	A=0.9999 / C=0.0001	A=1.00 / C=0.00	A=1.00 / C=0.00	[49]
rs121908073	366	DFNB7/11	*TMC1*: c.100C>T-p.Arg34Ter	3.28	1075 - 1900	Saudi Arabia	C=0.9999 / T=0.0001	C=1.0000 / T=0.00	C=1.000 / T=0.00	[51]
rs181949335	150	DFNB8	*TMPRSS3*: c.916G>A-p.Ala306Thr	1.98	NA	China	C=0.999718 / T=0.000282	C=1.00 / T=0.00	C=0.9997 / T=0.0003	[52]
rs80356605	23	DFNB9	*OTOF:* c.5816G>A-p.Arg1939Gln	43.5	NA	Japan	C=0.99993 / A=0.00, T=0.00007	C=1.0000 / A=0.00, T=0.00	C=1.00 / A=0.00, T=0.00	[53]
rs121908354	128	DFNB12	*CDH23*: c.C719T-p.Pro240Leu	3.10	NA	South Korea	C=1.00 / T=0.00	C=1.00 / T=0.00	C=0.9994 / T=0.0006	[54]
rs143797113	169	DFNB29	*CLDN14*: c.488C>T-p.Ala163Val	NA	NA	Newfoundland, Canada	G=0.999203 / A=0.000797	G=0.9998 / A=0.0002	G=1.00 / A=0.00	[56]
rs142846225	11	DFNB29	*CLDN14*: c.414G>A-p.Trp138Ter	63.6	NA	Yemen	C=1.00000 / T=0.00	C=0.9997 / T=0.0003	C=1.00 / T=0.00	[57]
rs74315437	NA	DFNB29	*CLDN14*: c.254T>A-p.Val85Asp	2.25	NA	Pakistan	A=1.0000 / C=0.00, T=0.00	A=1.00 /C=0.00, T=0.00	A=1.00 / C=0.00, T=0.00	[55]

rs200664140	NA	DFNB57	*PDZD7*: c.490C>T-p.Arg164Trp	NA	NA	South Korea	G=1.00 / A=0.00	G=1.0 / A=0.0	G=1.00 / A=0.00	[58]
rs367688416	27	DFNB59	*PJVK:* c.406C>T-p.Arg136Ter	28.5	150	Israel	C=0.99991 / T=0.00009	C=0.9997 / T=0.0003	C=1.00 / T=0.00	[3]
rs80338940	23	DFNBA1	*GJB2*: IVS1+1G>A (c.-23+1G>A)	NA	6000	Russia	C=0.99979 / T=0.00021	C=1.00 / T=0.00	C=1.00 / T=0.00	[61]
rs80338940	6	DFNBA1	*GJB2*: IVS1+1G>A (c.-23+1G>A)	21.4	1825-4100	Russia	C=0.99979 / T=0.00021	C=1.00 / T=0.00	C=1.00 / T=0.00	[39]
rs80338940	86	DFNBA1	*GJB2*: IVS1+1G>A (c.-23+1G>A)	79.34	800	Russia	C=0.99979 / T=0.00021	C=1.00/ T=0.0000	C=1.000 / T=0.00	[59]
rs80338943	174	DFNBA1	*GJB2*: IVS1+1G>A (c.-23+1G>A)	4.9	NA	Turkey	C=0.99979 / T=0.00021	C=1.00 / T=0.00	C=1.00 / T=0.00	[60]
rs80358272	287	DFNA2	*KCNQ4*: c.211delC-p.Gln71Ter	4.53	NA	Japan	NR	NR	NR	[62]
rs1064797088	39	DFNA3A	*GJB2*: c.136G>A-p.Asp46Asn	NA	NA	Iran	None	None	None	[50]
rs727505273	26	DFNA5	*GSDME*: c.991-15_991-13delTTC	NA	NA	China	GAAGAAGAAG=0.99994 / GAAGAAG=0.00006	GAAGAAGAAG=1.00 / GAAGAAG=0.00	GAAGAAGAAG=1.00 / GAAGAAG=0.00	[63]
rs727505273	14	DFNA5	*GSDME*: c.991-15_991-13delTTC	NA	NA	Korea	GAAGAAGAAG=0.99994 / GAAGAAG=0.00006	GAAGAAGAAG=1.0000 / GAAGAAG=0.0000	GAAGAAGAAG=1.00 / GAAGAAG=0.00	[64]
rs727505273	65	DFNA5	*GSDME*: c.991-15_991-13delTTC	NA	NA	Japan	GAAGAAGAAG=0.99994 / GAAGAAG=0.00006	GAAGAAGAAG=1.00 / GAAGAAG=0.00	GAAGAAGAAG=1.00 / GAAGAAG=0.00	[65]
rs727505273	11	DFNA5	*GSDME*: c.991-15_991-13delTTC	NA	NA	European	GAAGAAGAAG=0.99994 / GAAGAAG=0.00006	GAAGAAGAAG=1.00 / GAAGAAG=0.00	GAAGAAGAAG=1.000 / GAAGAAG=0.000	[66]
rs757172581	87	DFNA10	*EYA4*: c.1177C>T-p.Gln393Ter	NA	NA	South Korea	C=1.00000 / T=0.00	C=0.9997 / T=0.0003	C=1.000 / T=0.000	[54]
rs138527651	11	DFNA36	*TMC1*: c.1939T>C-p.Ser647Pro	34.0	NA	Morocco & Israel	T=0.99996 / C=0.00004	T=0.9998 / C=0.0002	T=1.00 / C=0.00	[67]

rs200171616	17	DFNA36	*TMC1*: c.1534C>T-p.Arg512Ter	77.7	NA	Finland	C=0.999929 / T=0.000071	C=1.00 / T=0.0	C=1.00 / T=0.000	[68]

rsID = Reference sequence ID; NR = Not reported; NA = Not available; Ref/Alt = Reference / Alternate Frequency; N = Sample size; Freq. = Frequency; yrs = Years; Ref. = Reference
