# Supplementary material for: Global Distribution of Founder Variants Associated with Non-Syndromic Hearing Impairment
Source: Genes (Basel). 2023 Feb 3;14(2):399. doi: 10.3390/genes14020399 (PMC9957346; doi:10.3390/genes14020399)
Supplement: Supplementary file 1 [file genes-14-00399-s001.zip › Table S2.pdf]

Table S2. Risk of bias assessment results

Study Reference	Risk of bias tool										
	**External Validity**				**Internal Validity**						**Score**
	1	2	3	4	5	6	7	8	9	10	11
[1]	Y	Y	N	NA	Y	Y	Y	Y	N	Y	7
[3]	Y	Y	Y	NA	Y	Y	Y	Y	N	Y	7
[11]	Y	Y	N	NA	Y	Y	Y	Y	N	Y	7
[24]	N	N	N	NA	Y	Y	Y	Y	N	Y	5
[25]	Y	Y	N	NA	Y	Y	Y	Y	N	Y	7
[26]	Y	Y	N	NA	Y	Y	Y	Y	NA	Y	7
[27]	Y	Y	N	NA	Y	Y	Y	Y	N	Y	7
[28]	Y	Y	N	NA	Y	Y	Y	Y	N	Y	7
[29]	Y	Y	N	NA	Y	Y	Y	Y	N	Y	7
[30]	Y	Y	N	NA	Y	N	Y	Y	N	Y	6
[31]	Y	Y	N	NA	Y	Y	Y	Y	N	Y	7
[32]	Y	Y	N	NA	Y	Y	Y	Y	N	Y	7
[33]	Y	Y	N	NA	Y	Y	Y	Y	N	Y	7
[34]	N	N	N	NA	Y	Y	Y	Y	N	Y	5
[35]	Y	Y	N	NA	Y	Y	Y	Y	N	Y	7
[36]	Y	Y	N	NA	Y	Y	Y	Y	N	Y	7
[37]	Y	Y	N	NA	Y	Y	Y	Y	N	Y	7
[38]	Y	Y	N	NA	Y	Y	Y	Y	N	Y	7
[39]	N	N	N	NA	Y	Y	Y	Y	N	Y	5
[40,41]	Y	Y	N	Y	Y	Y	Y	Y	Y	Y	9
[43]	Y	Y	N	NA	Y	Y	Y	Y	N	Y	7
[44]	Y	Y	N	NA	Y	Y	Y	Y	NA	Y	7
[45]	Y	Y	N	NA	Y	Y	Y	Y	N	Y	7
[46]	N	N	N	NA	Y	Y	Y	Y	N	Y	5
[47]	Y	Y	N	Y	Y	Y	Y	Y	Y	Y	9
[49]	Y	Y	N	NA	Y	Y	Y	Y	N	Y	7
[50]	N	Y	N	Y	Y	Y	Y	Y	Y	Y	8
[51]	Y	Y	N	Y	Y	Y	Y	Y	Y	Y	9
[52]	Y	Y	N	NA	Y	Y	Y	Y	NA	Y	7
[53]	Y	Y	N	NA	Y	Y	Y	Y	N	Y	7
[54]	Y	Y	N	NA	Y	Y	Y	Y	N	Y	7
[55]	N	N	N	NA	Y	Y	Y	Y	N	Y	5
[56]	Y	Y	N	Y	Y	Y	Y	Y	Y	Y	9
[57]	N	Y	N	Y	Y	Y	Y	Y	Y	Y	8
[58]	N	N	N	NA	Y	Y	Y	Y	N	Y	5
[59]	Y	Y	N	NA	Y	Y	Y	Y	NA	Y	7
[60]	Y	Y	N	NA	Y	Y	Y	Y	N	Y	7
[61]	N	Y	N	Y	Y	Y	Y	Y	Y	Y	8
[62]	Y	Y	N	NA	Y	Y	Y	Y	NA	Y	7
[63]	N	N	N	Y	Y	Y	Y	Y	NA	Y	6
[64]	N	N	N	NA	Y	Y	Y	Y	N	Y	5
[65]	Y	Y	N	NA	Y	Y	Y	Y	N	Y	7
[66]	N	Y	N	NA	Y	Y	Y	Y	N	NA	6
[67]	Y	Y	N	Y	Y	Y	Y	Y	Y	Y	9
[68]	Y	Y	N	Y	Y	Y	Y	Y	Y	Y	9
[69]	Y	Y	N	Y	Y	Y	Y	Y	Y	Y	9
[73]	Y	Y	N	NA	Y	Y	Y	Y	N	Y	7
[99]	Y	Y	N	NA	Y	Y	Y	Y	NA	Y	8

Y = Yes, Low risk of bias, **N** = No, High risk of bias, **NA** = Data no available to score
